# Clinical Wide-Field Retinal Image Deep Learning Classification of Exudative and Non-Exudative Age-Related Macular Degeneration

**DOI:** 10.7759/cureus.17579

**Published:** 2021-08-30

**Authors:** Nathaniel Tak, Akshay J Reddy, Juliette Martel, James B Martel

**Affiliations:** 1 Ophthalmology, California Northstate University College of Medicine, Elk Grove, USA; 2 Opthalmology, California Northstate University College of Medicine, Elk Grove, USA; 3 Health Sciences, California Northstate University, Rancho Cordova, USA

**Keywords:** artificial intelligence in medicine, macular degeneration, optos imaging, convolutional neural networks (cnn), computer-aided diagnosis

## Abstract

Background: Age-related macular degeneration (AMD) is a disease that currently affects approximately 196 million individuals and is projected to affect 288 million in 2040. As a result, better and earlier detection methods for this disease are needed in an effort to provide a higher quality of care. One way to achieve this is through the utilization of machine learning. A deep neural network, specifically a convoluted neural network (CNN) can be trained to differentiate between different types of AMD images given the proper training data.

Methods: In this study, a CNN was trained on 420 Optos wide-field retinal images for 70 epochs in order to classify between exudative and non-exudative AMD. These images were obtained and labeled by ophthalmologists from the Martel Eye Clinic in Rancho Cordova, CA.

Results: After completing the study, a model was created with 88% accuracy. Both the training and validation loss started above 1 and ended below 0.2. Despite only analyzing a single image at a time, the model was still able to accurately identify if the individual had AMD in both eyes or one eye only. The model had the most trouble with bilateral non-exudative AMD. Overall the model was fairly accurate in the other categories. It was noted that the neural network was able to further differentiate from a single image if the disease is present in left, right, or both eyes. This is a point of contention for further investigation as it is impossible for the artificial intelligence (AI) to extrapolate the condition of both eyes from only one image.

Conclusion: This research fostered the development of a CNN that was able to differentiate between exudative and non-exudative AMD. As well as determine if the disease is present in the right, left, or both eyes with a relatively high degree of accuracy. The model was trained on clinical data and can theoretically be used to classify other clinical images it has never encountered before.

## Introduction

Age-related macular degeneration is a chronic disease occurring in the central retina that affects 196 million people worldwide [1]. As the disease progresses, it leads to a loss of the visual field. This disease has a range of symptoms such as visual distortions and reduced central vision [1]. Typically, visual loss occurs in the later stages of the disease due to neovascularization and geographic atrophy [2]. As a result, age-related macular degeneration (AMD) can be further classified into exudative (wet) and non-exudative (dry). Wet AMD occurs when the choroidal neovascular membranes under the retina leak fluid and blood. If wet AMD is caught in the early stages it can be treated with anti-vascular endothelial growth factor (anti-VEGF) shots [3]. This, in turn, damages the retina. Dry AMD occurs when the retina and choroid degrade due to atrophy or detachment of the retinal pigment epithelium [3]. In the field of ophthalmology, machine learning has been extensively utilized for classifying diseases such as glaucoma and diabetic retinopathy with high degrees of success [4-5]. Even with the utilization of teleophthalmology, machine learning has the potential to vastly improve the quality of care provided [6]. Convoluted neural networks (CNNs) composed of convolutional layers are primarily used for visual applications. This can be a form of a deep neural network as there are often times many hidden layers. This study was conducted in order to develop a deep learning model that is able to differentiate between exudative and non-exudative AMD through analyzing wide-field images from a clinical setting. This will be further differentiated between AMD in the left, right, and both eyes.

## Materials and methods

This was a retrospective cohort study of individuals diagnosed with exudative or non-exudative macular degeneration. Retinal fundus images from patients and diagnoses by ophthalmologists were obtained from the Martel Eye Clinic in Rancho Cordova, CA. 

Images used were wide-field Optos images collected from 2017 to 2021. Images collected were initially filtered to remove low-quality images from the dataset. They were then labeled based on the respective side they were taken on (left or right) and ICD-10-CM diagnostic codes (H35.313, H35.323, H35.322, H35.321, H35.312, H35.311, or H35.35). The images labeled with H35.35 were relabeled to “#na#”. From an initial pool of 957 images, 20% of the images and their labels were randomly sampled and separated into the validation set. The remaining images were randomly sampled until each category had a total of 50 images each. This created a training set of 350 images and a validation set of 70 images for a total of 420 images used. The patient sample size for this study was 210. 

The machine-learning CNN algorithm was run on a Windows computer with an Nvidia GTX 3090 24GB. A batch size of four images was used with an initial learning rate of 1 x 10-3. Batch image augmentations were applied along with resizing of the images to 1950 x 1535 pixels. The CNN was run for a total of 70 epochs taking about three minutes and 20 s for each epoch.

## Results

**Figure 1:**
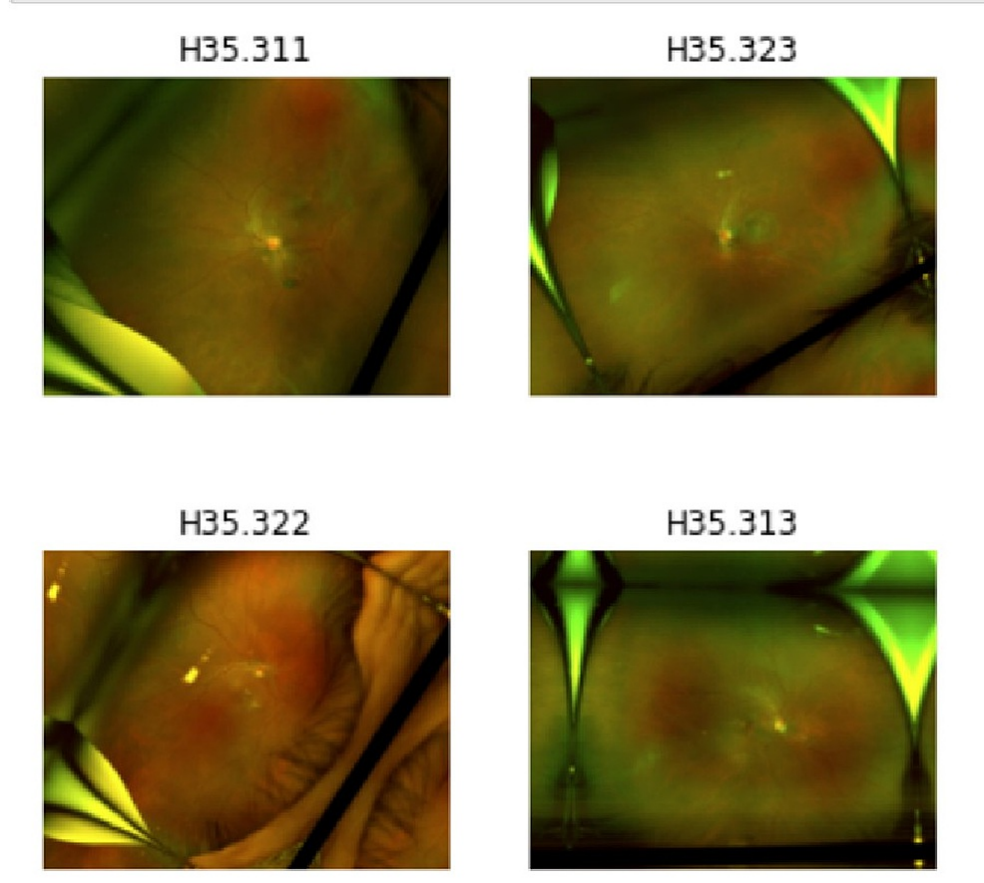
Images with ICD-10 labels and transformations.

Figure 1 shows a sample of four images with transformations applied along with their ICD-10 diagnostic code. These images after the transformations are the ones that are used to train the model. Since these images were obtained from a clinical setting there is a significant variation in the quality of images. This increased the difficulty of training the model requiring more images in order to compensate for the variations in each image. Image augmentations allow for new data to be created by modifying the original image in order to make an image the model has not been trained on. 

**Figure 2:**
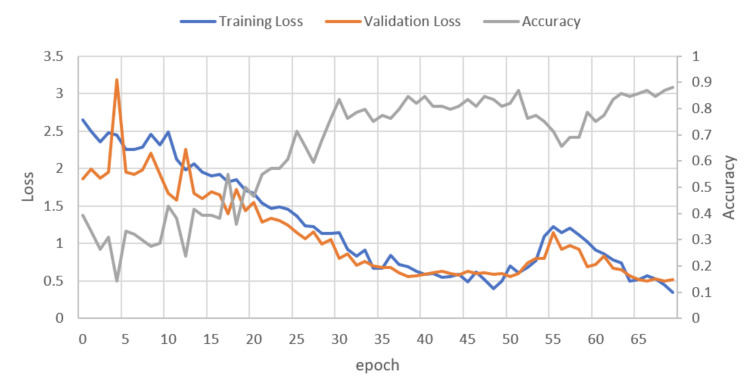
A plot of training loss, validation loss, and accuracy per epoch.

Figure 2 shows how the training loss, validation loss, and accuracy progressed for each epoch. It was determined that the CNN model had a final accuracy of 88%. Overall there was a general trend of decreasing loss for both the validation and training set as the number of epochs increased. This trend was opposite to accuracy as it increased as the number of epochs increased. There was a dip in accuracy around epoch 56. This may be due to the model getting stuck in local minimum.

**Figure 3:**
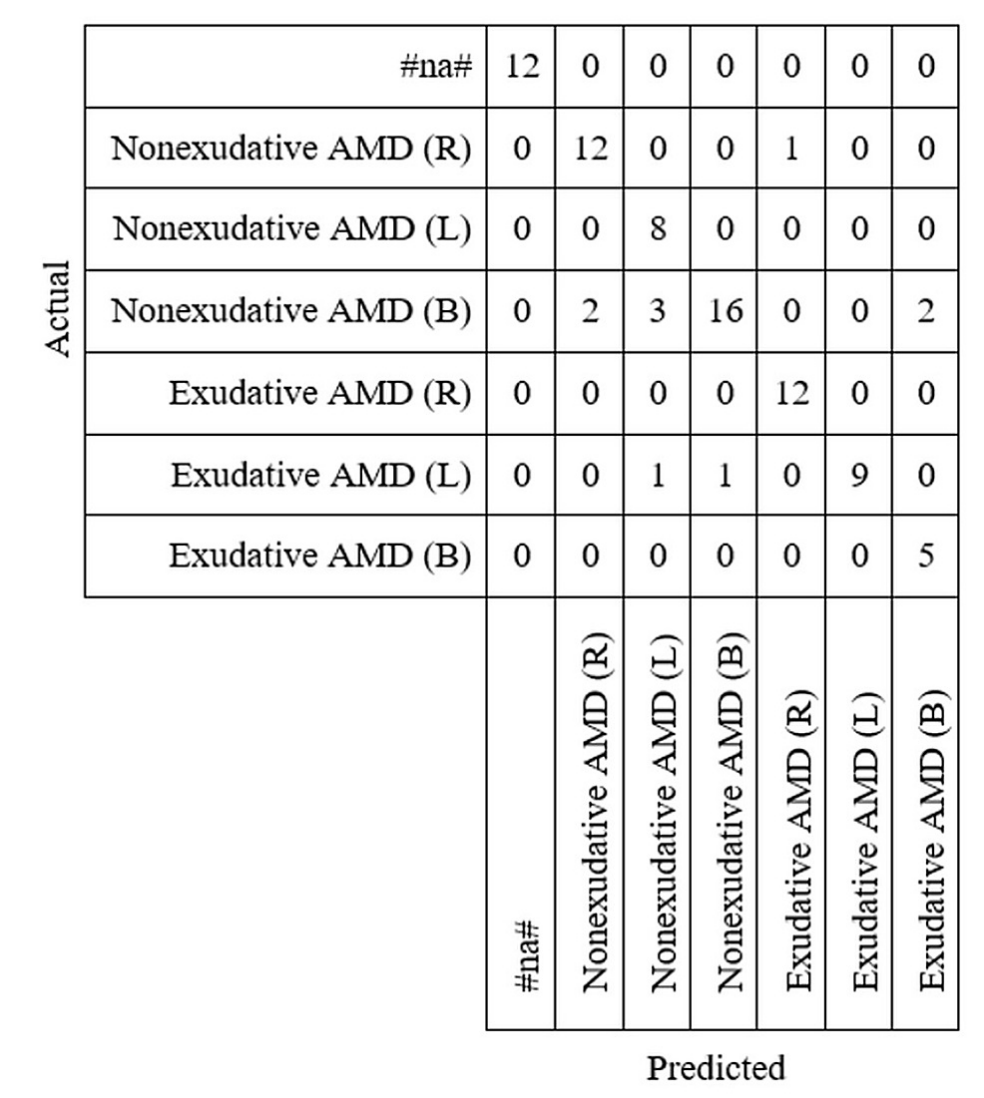
Confusion matrix between actual and predicted categories of the validation dataset.

Figure 3 shows a confusion matrix that displays the category and predicted category of the validation dataset. This is the result of testing the model on the validation dataset. Overall, the model predicted accurately when compared to the actual diagnosis. The model had relatively little difficulty when determining if the image was a right or left side image when compared to bilateral images.

## Discussion

Age-related macular degeneration is an extremely pervasive ocular disease that can cause complications such as blindness if left untreated [7]. Macular degeneration can also lead to deterioration of the fovea which can cause decreased color perception [8]. The two primary types of macular degeneration are exudative and non-exudative [8-9]. Exudative macular degeneration, which is less common, occurs when there is a leakage of fluid from new blood vessels that formed below the blood vessels. This leakage of fluid causes the macula to be distorted from its original position which results in poorer central vision. Non-exudative macular degeneration is where the macula degenerates and thins over time due to the natural aging process [8-9]. More commonly, people have non-exudative macular degeneration which can be treated with laser photocoagulation [10]. In fact, over 8.7% of the world’s population becomes blind due to macular degeneration [11]. Macular degeneration is a treatable disease for most people if it is caught early enough. There are many features on retinal scans, such as drusen, that physicians can look at to determine if a patient has macular degeneration; however, many individuals do not receive treatment or a diagnosis on time either due to negligence or more commonly a lack of access to proper medical care [12]. With an increasing patient population, there will always be a shortage of physicians to treat diseases such as macular degeneration. In order to counterbalance this issue, technology needs to be improved to enable patients to access quality care at a faster rate. Fortunately, recent advances in technology have produced artificial intelligence (AI) that can make tasks such as diagnosis more efficient. If AI could be used to assist physicians with diagnosis, then responses to clinical issues would be more efficient and accurate. AI has been already proven to be capable of diagnosing ocular diseases such as diabetic retinopathy [5,13]. The abilities of an AI software is limited by the information and data biases within its training set and as such should still be checked by an attending physician when in use [14]. Although it may not be possible to produce a completely accurate AI software, it may be possible to develop software that is accurate enough to help physicians diagnose cases at a faster rate. In this investigation, the authors attempt to develop an AI software that can be utilized to diagnose cases where patients have exudative or non-exudative macular degeneration in either their left eye, their right eye, or both eyes. Our investigation builds upon previous studies that have demonstrated how AI software can accurately diagnose macular degeneration within a patient [15-20]. 

In this investigation, the AI software was trained for 70 epochs utilizing 350 retinal scans of patients with either exudative and non-exudative macular degeneration. The AI software was able to distinguish patients who had exudative macular degeneration in their left eye and bilaterally with an accuracy of 100%. It was also able to determine if a patient had exudative macular degeneration in their right eye with an accuracy rating of 81.8%. The software was also able to determine if a patient had non-exudative macular degeneration in their right eye with 100% accuracy, their left eye with 92.3% accuracy, and both eyes with 69.6% accuracy. The program was also able to determine if a patient did not fall into any of the other categories with an accuracy rating of 100%. The results from the data suggest that it may be easier for the AI software to identify signs of exudative macular degeneration. This could potentially illustrate that there are more obvious signs on retinal scans for cases of exudative macular edema such as physicians that make it easier for the AI to obtain higher accuracy results to detect the disease. This could also indicate that the retinal scans for exudative macular degeneration may have been of better quality, or that the limited sample size resulted in these accuracy readings. The accuracy ratings of the AI software for detecting macular degeneration bilaterally were surprisingly high. Since only a single image is processed at a time, the program should have had difficulty determining if the image bilateral, left, or right as bilateral contains both left and right images. Despite this, the majority of the bilateral images were able to be correctly predicted. This would suggest that there is a difference in the macular degeneration between bilateral cases than cases only in the left or right eye. This would be a point of further research to determine if there is a fundamental difference between bilateral macular degeneration and macular degeneration only occurring in one eye.

Our findings have crucial implications for the field of medicine. Our report is the first to our knowledge that reports an AI software that is able to scan a single retinal image and tell if a patient has macular degeneration in both eyes. This proves that the application and usage of AI software in the field of medicine is not only useful at decreasing costs and improving patient outcomes but that it can also be utilized to identify new patterns and make discoveries within the field of medicine. One of the biggest strengths of this study is that the AI software utilized raw unprocessed clinical data to identify patterns and produce results. Unlike previous studies that were done using processed images and datasets, our software was able to function using low-quality images [12-15, 20]. This means that our AI will be more applicable to the practical clinical setting if physicians decide to use our tool. Although our investigation produced fairly accurate results, there were some limitations to the study. One of the main limitations to this study was that the patient data collected for the AI software all came from a patient database located in one city. Another limitation to our study was the sample size that we used for both our training and validation sets. The training set for this study was the image database that was used to train the AI software to recognize patterns within the retinal scans to detect macular degeneration. The validation set was the image database used to verify the accuracy and ability of the AI software to detect macular degeneration within retinal scans. Had we used more images from the clinical database for both of these sets, the accuracy of the software to diagnose patients with either exudative or non-exudative macular degeneration could have dramatically increased. The results could have differed if we utilized a database of images that took patient data from multiple areas across the world. Further research on the application of AI in a clinical setting needs to be conducted in order to increase the accuracy and the generalizability of the results that are produced from the technology.

## Conclusions

The authors in this investigation developed a CNN algorithm that is able to differentiate between exudative and non-exudative macular degeneration from ultrawide field Optos images. On the validation dataset, the model was calculated to be 88%. The model was able to differentiate from analyzing a single image if AMD is present in the left eye, right eye, or both eyes. Being able to identify if AMD is present in both eyes from an image of a single retina. AMD needs to be caught early on in order to prevent further patient complications, therefore, it is quintessential for physicians to continue developing tools and methods that facilitate diagnosis. Further research is required to verify and explain the possibility of extrapolating the fact that a patient has AMD in both eyes when only looking at one retinal scan. The results from this exploration demonstrate that AI has the potential to have a significant impact on diagnostics and the overall quality of care in the healthcare industry.
